# Transcriptional Regulators of T Helper 17 Cell Differentiation in Health and Autoimmune Diseases

**DOI:** 10.3389/fimmu.2020.00348

**Published:** 2020-03-12

**Authors:** Alessia Capone, Elisabetta Volpe

**Affiliations:** ^1^Neuroimmunology Unit, IRCSS Fondazione Santa Lucia, Rome, Italy; ^2^Department of Biology and Biotechnology Charles Darwin, Sapienza University, Rome, Italy

**Keywords:** T helper 17 cells, interleukin-17, retinoic acid receptor related orphan nuclear receptor γt, multiple sclerosis, Crohn's disease, rheumatoid arthritis, psoriasis

## Abstract

T helper (Th) 17 cells are a subtype of CD4 T lymphocytes characterized by the expression of retinoic acid-receptor (RAR)-related orphan receptor (ROR)γt transcription factor, encoded by gene *Rorc*. These cells are implicated in the pathology of autoimmune inflammatory disorders as well as in the clearance of extracellular infections. The main function of Th17 cells is the production of cytokine called interleukin (IL)-17A. This review highlights recent advances in mechanisms regulating transcription of IL-17A. In particular, we described the lineage defining transcription factor RORγt and other factors that regulate transcription of *Il17a* or *Rorc* by interacting with RORγt or by binding their specific DNA regions, which may positively or negatively influence their expression. Moreover, we reported the eventual involvement of those factors in Th17-related diseases, such as multiple sclerosis, rheumatoid arthritis, psoriasis, and Crohn's disease, characterized by an exaggerated Th17 response. Finally, we discussed the potential new therapeutic approaches for Th17-related diseases targeting these transcription factors. The wide knowledge of transcriptional regulators of Th17 cells is crucial for the better understanding of the pathogenic role of these cells and for development of therapeutic strategies aimed at fighting Th17-related diseases.

## Introduction

T helper (Th) 17 cells are a subtype of CD4 T lymphocytes, specialized in immune response against fungi and some extracellular bacteria (1–4). The interleukin (IL)-17A, originally named CTLA8, is the most representative cytokine produced by Th17 cells (3, 5, 6), also produced by cytotoxic T lymphocytes, and innate lymphocytes, including γδ T, natural killer T, and group 3 innate lymphoid cells (7).

The binding of IL-17A with its receptor activates the target cells, such as epithelial cells, endothelial cells, and fibroblasts (3, 4, 8) and induces CXCL1, CXCL2, and CXCL8, which attract myeloid cells such as neutrophils to the infected or injured tissue (9); IL-6 and G-CSF, which promote myeloid-driven innate inflammation (10); and β-defensins, S100A8, and lipocalin 2, which protect the host during acute microbial invasion (11).

In addition to IL-17A, Th17 cells produce IL-17F, IL-21, IL-22, and, in human, also IL-26 (3, 5, 6, 12), which collectively ensure an appropriate defense against pathogens. In fact, genetic defects in the Th17–cytokine pathways lead to severe mucocutaneous candidiasis (13–15).

However, a dysregulated activity of Th17 cells has been associated to autoimmune diseases, such as multiple sclerosis (MS), rheumatoid arthritis, psoriasis, and Crohn's disease (8, 16).

Given the relevance of Th17 cells in both physiological and pathological contexts, numerous studies investigated the molecular mechanisms regulating the transcriptional program of Th17 cells.

Majority of the Th17 transcription factors were discovered and validated through analysis of IL-17A expression in mice deficient for specific transcription factors, and mice containing a GFP reporter cDNA knocked-in at the site for initiation of the translation of specific transcription factors (17–21). Similarly, the *in vitro* expression of IL-17A was assessed in cells cotransfected with constructs overexpressing the specific transcription factors and reporter constructs containing regions upstream of the *Il17a* transcription start site (17, 19). More recently, modern technologies, such as chromatin immunoprecipation (ChIP) and single-cell RNA-sequencing, were allowed to better explore the functions of transcription factors in Th17 cells (22–24). However, although the expression of Th17 transcription factors was validated in human Th17 cells, most of the studies demonstrating their regulatory mechanism were performed in murine cells.

The first transcription factor discovered, designated as the “lineage defining transcription factor of Th17 cells,” is RORγt, which is essential and sufficient to induce Th17 lineage fate in both human and mouse cells (5, 17, 25).

However, succeeding studies revealed that multiple transcriptional regulators contribute to full Th17 differentiation program through several mechanisms, including binding to specific regions of *Il17a* and *Rorc* genes, or interacting and synergizing with RORγt, or facilitating the recruitment of other proteins on *Il17a* or *Rorc* promoters.

Collectively, Th17 transcriptional regulators may contribute to Th17 functions in physiological and pathological contexts. Thus, in this review, we reported recent advances on the molecular mechanisms directly regulating transcription of *Il17a* and *Rorc*. Moreover, we discussed their involvement in autoimmune disorders associated to an exaggerated Th17 response. Finally, we discussed the recent therapeutic approaches targeting Th17 transcriptional regulators in Th17-related autoimmune diseases.

## Retinoic Acid-Receptor-Related Orphan Receptor (ROR) Transcription Factors in Th17 Cells

The retinoic acid-related orphan nuclear receptors (RORs) belong to a superfamily of ligand regulated transcription factors (26, 27). ROR transcription factors bind DNA response elements, called ROR response elements (ROREs) (26, 28), consisting of the consensus core motif AGGTCA preceded by a 5′ A/T-rich sequence located into regulatory regions of target genes (27).

The interaction of ROR factors with their specific ligands allows recruitment of cofactor proteins, which leads to the transcription of their target genes (29).

ROR family is composed of three members, RORα (NR1F1), RORβ (NR1F2), and RORγ (NR1F3) (30–32), encoded by *Rora, Rorb*, and *Rorc* genes, respectively. *Ror* genes may encode different protein isoforms, among which RORα4 and RORγt are the unique isoforms expressed in cells of the immune system (29).

Interestingly, RORγt is expressed in thymocytes at the double-positive stage of T cell development, but is absent in mature thymocytes and in mature naive T cells in spleen and peripheral lymph nodes (33). In 2006, RORγt has been detected in IL-17-producing T cells (17), and it has been shown to play a central role in Th17 differentiation (17, 34).

Precursors or derivatives of cholesterol, such as desmosterol (35) and oxysterols (36), respectively, have been identified as activator ligands of RORγt, while bile acid synthesized from cholesterol called 3-oxoLC is an inhibitory ligand of RORγt (37).

**Figure 1:**
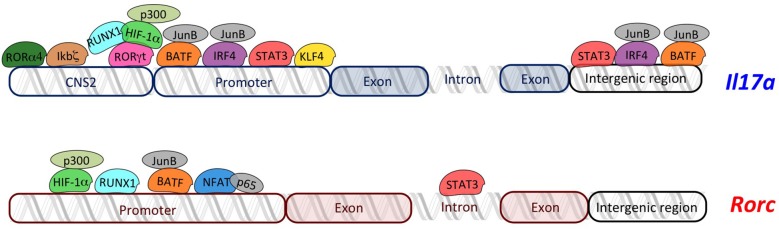
Overview of transcriptional regulators of *Il17a* and *Rorc*. The transcriptional regulators of Th17 cells (RORγt, RORα4, ikbζ, RUNX1, HIF-1α, STAT3, IRF4, NFAT, KLF4, and BATF) regulate transcription of *Il17a* and *Rorc* by binding specific regions in their loci. Schema does not respect the real organization and structure of each gene locus.

RORγt regulates *Il17a* transcription by binding RORE sequences present in the 2-kb promoter fragment upstream of the transcription start site (38). In addition, the conserved non-coding sequences (CNS)2 (also called CNS5) located in the vicinity of the *Il17a* gene (approximately 5-kb upstream of promoter) (39) contains two ROREs, which are also conserved in human (39, 40). It has been demonstrated that RORγt binds CNS2 of the *Il17a* gene (Figure 1) and mediates *Il17a* transcription by controlling the chromatin remodeling. In fact, CNS2 is also bound by p300 and JmjC domain-containing protein (JMJD)3 that mediate permissive histone acetylation (41, 42) and remove repressive histone marker H3K27me3 (43–45), respectively, resulting in hyperacetylation of histone H3 (46, 47). Moreover, CNS2 interacts with *Il17a* promoter by forming a loop, and brings CNS2-associated histone remodeling enzymes to the promoter for the activation of *Il17a* transcription (39).

Similarly, it has been demonstrated that RORα4 overexpression promotes, while RORα4 deficiency impairs, *Il17a* expression (40). Interestingly, coexpression of RORα4 and RORγt causes the synergistic increase in IL-17A, indicating that RORα4 and RORγt work together to regulate Th17 cell differentiation (40, 48).

Given the high similarity of DNA-binding domains between RORα4 and RORγt, they activate *Il17a* transcription through the same molecular mechanism (40) (Figure 1).

However, RORα4 and RORγt are not sufficient to generate and specify the full Th17 program.

In fact, transcriptional regulators of RORγt, as well as other transcription factors that interact with RORγt, or bind the promoter or the intergenic regions of the *Il17a* locus, play a crucial role in the generation of Th17 cells.

## Other Transcriptional Regulators of *Rorc* and *Il17A*

The transcription of RORγt is initiated by activation of the promoter RORC2 into the *Rorc* locus. RORC2 promoter contains nuclear factor of activated T cells (NFAT)-binding sequences, specific for NFAT and nuclear factor (NF)-kB proteins. Recently, it has been reported that the p65 NF-kB subunit and NFATc2 bind human *Rorc* promoter and promote a permissive chromatin conformation at RORC2 regulatory regions (49). Consistently, it has been reported that two NF-kB proteins, c-Rel and p65, activate the murine *Rorc* promoter (50).

Interestingly, the nuclear protein inhibitor of kB (IκB)ζ, which belongs to the IkB kinases and regulates activation of NF-kB pathway, binds CNS2 elements in *Il17a* locus (Figure 1), thus leading to an efficient recruitment of transcriptional coactivators with histone acetylase activity (18) and promoting *Il17a* expression without modulating expression of *Rorc* and *Rora* (51, 52).

CNS2 region of *Il17a* is bound by another transcriptional regulator called Runt-related transcription factor (RUNX)1, whose effect is dependent on RORγt. In fact, it has been demonstrated that RUNX1 interacts with RORγt to potentiate *Il17a* expression and is required for the full effect of RORγt on *Il17a* expression (38) (Figure 1). Additionally, RUNX1 plays a role in Th17 differentiation, independently of RORγt, by binding the promoter of the gene encoding RORγt through three conserved RUNX1-binding sites (53) (Figure 1).

Hypoxia-inducible factor (HIF)-1α is a key metabolic sensor (19, 54), which binds hypoxia response element (HRE, a conserved HIF)-1α-binding site) located in the proximal region of the *Rorc* promoter, in both human and mouse (19). Moreover, HIF-1α might physically associate with RORγt, serving as a coactivator for RORγt, thus contributing to *Il17a* expression without direct DNA binding on *Il17a* locus (19) (Figure 1). Further studies discovered that HIF-1α activates target genes by recruiting the factor p300, which possesses histone acetyltransferase activity and acetylates histones to “open” the chromatin structure (55). Indeed, the colocalized binding of RORγt, HIF-1α, and p300 occurs at the promoter of the *Il17a* gene (19).

Signal transducer and activator of transcription (STAT) 3 is another transcription factor regulating RORγt, and IL-17A (56) by interacting with the Stat-binding domains into the *Rorc* first intron, the *Il17a* promoter, and the intergenic region of the *Il17a* locus (Figure 1) (56–58). Moreover, STAT3 regulates positive epigenetic modifications, increasing permissive H3K4me3 marks on its target genes, including *Rorc, Rora*, and another gene encoding for transcriptional regulator of Th17 cells, called basic leucine zipper ATF-like transcription factor (BATF) (56).

BATF forms a heterodimer with JunB, and binds to the *Il17a* promoter as well as two conserved intergenic elements in the *Il17a* locus in Th17 cells (Figure 1). Interestingly, BATF synergizes with RORγt by binding to an overlapped conserved region recognized by RORγt into *Il17a* gene (20). Furthermore, the complex JunB and BATF also promotes the transcription of *Rorc* and *Rora* (58, 59) (Figure 1).

Genome-wide JunB-DNA binding analysis, using ChIP sequencing with anti-JunB antibody, revealed that JunB colocalizes in Th17 cells with another transcription factor, called interferon regulatory factor (IRF)4, involved in Th17 differentiation (21). In fact, IRF4 targets sequences enriched for activating protein 1 (AP-1)–IRF composite elements (AICEs) located into regulatory elements of the *Il17a* promoter (58, 60), which are cobound by BATF, an AP-1 factor (61). Thus, IRF4 and BATF bind cooperatively to structurally divergent AICEs to promote IL-17A activation in Th17 cells (61). Importantly, not only *Il17a* locus but also *Il21, Il22*, and *Il23r* loci contain one or more coincident binding peaks for IRF4 and BATF that were positioned in promoters and/or intronic regions, and ChIP assays verified the binding to these regions of both IRF4 and BATF complexed with JunB (61). The Kruppel-like factor (KLF)4 is another factor involved in the direct regulation of IL-17A, as demonstrated by ChIP analysis. In fact KLF4 binds the *Il17a* promoter and induces IL-17A expression, independently of RORγt (62).

Altogether, this information reveals a complex interconnected network of transcriptional regulators that finely regulates generation of Th17 cells.

The timing of transcriptional events leading to the full Th17 differentiation remains enigmatic. However, the transcriptional regulators activated upon T cell receptor engagement, such as NFAT, likely initiate the differentiation process by inducing RORγt transcription, and up-regulating receptor for polarizing cytokines, whose ligation leads to activation of other transcription factors. Among them, BATF, IRF4, and STAT3 are considered initiator transcription factors (24, 63). In fact, BATF and IRF4 are responsible of initial chromatin accessibility in *Il17a* locus and, with STAT3, of initiation of the transcriptional program that is then globally tuned by the lineage-specific transcription factor RORγt, which plays a pivotal deterministic role at key loci (24, 63). Then, RUNX1, HIF1a, and IκBζ can be considered cooperators of ROR nuclear receptors.

Importantly, there is high interconnectivity among transcription factors, including positive feedback loops reinforcing expression of initiator transcription factors BATF, IRF4, and STAT3 (24).

However, a negative feedback loop mediated by c-Maf, which is induced by initiator transcription factors, may limit Th17 response. In particular, c-Maf is a transcriptional regulator that, in Th17 cells, functions as a negative regulator, attenuating the expression of pro-inflammatory loci (e.g., *Batf*, *Rora, Runx1, Il1r1, Ccr6*, and *Tnf*) and positively regulating few loci linked to attenuating inflammation (e.g., *Il9, Il10, Lif*, and *Ctla4*). Another transcription factor known to limit Th17 response is Fosl2 exerting antagonistic effect to BATF, by competing for the same binding sites and by directly repressing BATF (24). STAT1 and STAT5 are known to inhibit Th17 polarization by directly binding *Rorc* or *Il17a* loci. In particular, STAT5 represses IL-17A induction by binding the *Il17a* locus, removing accessible histone marks, and displacing STAT3 occupancy (64, 65); STAT1 has been shown to bind upstream of the *Rorc* locus in human Hela cells (66).

## Th17-Related Transcriptional Regulators in Autoimmune Diseases

Given the crucial role of Th17 cells in autoimmune disorders, the altered expression of Th17 transcriptional regulators may be related to a persistent Th17 cell response typical of diseases, such as psoriasis, rheumatoid arthritis, Crohn's disease, and MS (16). The role of the transcription factors activating a Th17 response has been mainly investigated in the murine model of MS, the experimental autoimmune encephalomyelitis (EAE), where deletion of each specific Th17 transcription factor reduced the disease (17–21, 40, 62, 67, 68). However, the potential involvement of such transcription factors in human autoimmune diseases, as well as their expression in immune cells from patients, has not been largely investigated.

It has been reported that the levels of phosphorylated STAT3 (pSTAT-3) in lymphocytes are up-regulated in MS patients during relapse compared to healthy donors and MS patients in remission phase. Moreover, pSTAT-3 levels positively correlate with magnetic resonance imaging data, indicating that STAT3 activation is associated to disease activity (69). In contrast, the expression of RORγt analyzed at transcriptional (70) and protein level (71) does not differ between MS patients and healthy donors.

However, the activity of RORγt is ligand regulated and the putative natural ligands of RORγt are molecules of the cholesterol pathway. In this context, it has been reported that levels of oxysterols in relapsing-remitting MS patients were associated with conversion to secondary progressive-MS (72).

Moreover, an aberrant activation of STAT3 was found in intestinal T cells of Crohn's disease patients compared to healthy donors (73); the expression of IRF-4 was significantly increased in inflammatory cells of psoriasis patients than that in healthy controls (74); HIF-1α was found strongly expressed by immune cells in the intimal layer of the synovium in rheumatoid arthritis patients (75). However, the lack of correlations with clinical parameters in most part of these studies does not permit the definition of the role of the enhanced expression of those transcriptional regulators in human diseases.

Additionally, genetic abnormalities in Th17 transcriptional regulators may favor Th17 cell response and may influence susceptibility to autoimmune diseases. However, few studies demonstrate association between gene variants of Th17 transcription factors and Th17-related diseases. For instance, single-nucleotide polymorphisms (rs734232) affecting the consensus-binding site for RUNX1, or *Runx1* itself, are associated with susceptibility to rheumatoid arthritis and psoriasis (76–78), while *Stat3* gene was identified as risk locus for Crohn's disease and MS (79, 80).

## Therapeutic Approaches Targeting Transcriptional Regulators of Th17 Cells

Antibodies targeting IL-17A are approved for the treatment of psoriasis (81), while this approach is ineffective in MS, and deleterious in Crohn's disease (82). Recently, antagonists of Th17 transcriptional regulators have been proposed as potential new treatments of Th17-mediated diseases. Given the high cell specificity, RORγt is the transcription factor representing the ideal target for the manipulation of Th17 cell response. Several molecules targeting RORγt have been discovered and tested in murine models: digoxin, urosolic acid, and SR1001 reduce EAE severity (83–85); BI119 abrogates experimental colitis (86); SR2211 and JNJ-54271074 have therapeutic effect on experimental arthritis (87, 88); TMP778 and S18-000003 show efficacy in a psoriasis-like skin inflammation model (89, 90). In addition, other RORγt inverse agonists have been discovered (carbazole carboxamides, MG2778, TAK-828F, 6-substituted quinolines, A213) and tested as negative regulators of Th17 response (Table 1) (91–96).

**Table 1:** List of the therapeutic approaches targeting transcriptional regulators of Th17 cells.

**Compound**	**Target**	**Disease**	**Status**	**References**
Digoxin	RORγt	Multiple sclerosis	Mouse model	(81)
Urosolic acid	RORγt	Multiple sclerosis	Mouse model	(82)
SR1001	RORγt	Multiple sclerosis	Mouse model	(83)
BI119	RORγt	Colitis	Mouse model	(84)
SR2211	RORγt	Arthritis	Mouse model	(85)
JNJ-54271074	RORγt	Arthritis	Mouse model	(86)
A213	RORγt	Psoriasis	Mouse model	(91)
TMP778	RORγt	Psoriasis	Mouse model	(87)
S18-000003	RORγt	Psoriasis	Mouse model	(88)
Carbazole carboxamides	RORγt	Autoimmune disorders	*in-vitro* cell models	(90)
MG2778	RORγt	Autoimmune disorders	*in-vitro* cell models	(92)
TAK-828F	RORγt	Autoimmune disorders	*in-vitro* cell models	(93)
6-substituted quinolines	RORγt	Autoimmune disorders	*in-vitro* cell models	(94)
VTP-45489	RORγt	Psoriasis	To be tested in clinical trial	(95)
VTP-43742	RORγt	Psoriasis	Phase II terminated for liver toxicity	(95)
GSK-2981278	RORγt	Psoriasis	Phase II terminated	(95)
JTE-151	RORγt	Autoimmune disorders	Discontinued for further development	(95)
JNJ-3534	RORγt	Autoimmune disorders	Discontinued for further development	(95)
ABBV-553	RORγt	Psoriasis	Phase I terminated for safety concern	(95)
TAK-828	RORγt	Autoimmune disorders	Discontinued for further development	(95)
AZD-0284	RORγt	Autoimmune disorders	Discontinued for further development	(95)
ABBV-157	RORγt	Psoriasis	Phase I recruiting	(96)
JTE-451	RORγt	Psoriasis	Phase I Active, not recruiting	(96)
ESR-114	RORγt	Psoriasis	Phase I completed	(96)
ARN-6039	RORγt	Multiple Sclerosis	Phase I completed	(96)
AUR-101	RORγt	Psoriasis	Phase II active, not recruiting	(96)
RTA-1701	RORγt	Autoimmune disorders	Phase I completed	(96)
GSK2981278	RORγt	Psoriasis	Phase II completed	(96)
SAR-441169	RORγt	Psoriasis	Phase I	(96)
ROR antagonists	RORγt	Inflammatory diseases	Phase I	(96)
2-benzoyl-phenoxy acetamide	HIF-1α	Arthritis	Mouse model	(97)
STA-21	STAT3	Psoriasis	Phase II completed	(98)

Clinical studies testing the actual clinical efficacy and eventual side effects are active or completed. For instance, the oral compound VTP-43742 demonstrated efficacy through the reduction of clinical scores in psoriasis patients (NCT02555709). However, clinical data also showed liver toxicity, and VTP-43742 has been replaced with a new improved molecule VTP-45489 (Table 1). Similarly, other early clinical agents like GSK-2981278, JTE-151, JNJ-3534, ABBV-553, TAK-828, and AZD-0284 were either discontinued or suspended for further development (Table 1) (99). Currently, novel RORγt inhibitors are monitored in the clinical studies: ABBV-157 in psoriasis phase I (NCT03922607); JTE-451 and ESR-114 in psoriasis phase II (NCT03832738 and NCT03630939, respectively); ARN-6039 in MS phase I (NCT03237832); AUR-101 in psoriasis phase II (NCT04207801); RTA-1701 in healthy phase I (NCT03579030); GSK2981278 in psoriasis phase I (NCT03004846 and NCT02548052); SAR-441169 in psoriasis phase I; and ROR antagonists in inflammatory disease phase I (100) (Table 1).

Another promising target among Th17 transcription factors is HIF-1α. To date, the most advanced HIF pathway-targeted pharmaceuticals in terms of clinical development are cell-permeable prolyl hydroxylase inhibitors, evaluated for treatment of anemia. A number of HIF inhibitors have been developed also for cancer therapy (97) and are considered promising novel treatments for rheumatoid arthritis (101), such as the 2-benzoyl-phenoxy acetamide that acts as anti-arthritic agent in an experimental adjuvant induced arthritis rat model (98) (Table 1). However, none of the compounds targeting HIF-1α has been assessed in clinical trials for rheumatoid arthritis.

STAT3 is another potential drug target currently used for cancer therapy given its aberrant activation in many human tumors (102). Concerning Th17-related diseases, the small STAT3 inhibitor STA-21 has been tested on psoriasis patients in a nonrandomized study, and psoriatic lesions in six of the eight patients showed improvement after topical STA-21 treatment for 2 weeks (NCT01047943) (Table 1) (103). However, this effect is likely related to the inhibition of epidermal keratinocyte proliferation, rather than to immune cell activity (103).

Collectively, these data indicate that Th17 transcriptional regulators are promising targets for Th17-related diseases. However, given their broad expression in different cell types, it is crucial to develop inhibitors highly specific for immune cells to minimize off-target effects.

## Conclusions

Since the discovery of Th17 cells, remarkable advances in the understanding of Th17 response have been reported. In particular, the study of the mechanisms regulating the transcription of *Rorc* and *Il17a* genes has advanced our understanding of the generation of Th17 cells. Moreover, small molecules interfering with these mechanisms provide promising results in pre-clinical research and clinical trials. Future studies further detailing the transcriptional program of Th17 cells could lead to the identification of pathways or regulators that are specifically activated during diseases. Advances in these points are critical for the development of new compounds that target more accurately the pathogenic effect of Th17 cells, and that could become new therapeutic strategies in Th17-related diseases.

## Author Contributions

AC drafted the manuscript. EV critically reviewed the manuscript and finalized the manuscript for submission. AC and EV approved the final version.

### Conflict of Interest

The authors declare that the research was conducted in the absence of any commercial or financial relationships that could be construed as a potential conflict of interest.
